# Discovery of gene expression-based pharmacodynamic biomarker for a p53 context-specific anti-tumor drug Wee1 inhibitor

**DOI:** 10.1186/1476-4598-8-34

**Published:** 2009-06-08

**Authors:** Shinji Mizuarai, Kazunori Yamanaka, Hiraku Itadani, Tsuyoshi Arai, Toshihide Nishibata, Hiroshi Hirai, Hidehito Kotani

**Affiliations:** 1 Department of Oncology, Tsukuba Research Institute, Merck Research Laboratories, Banyu Pharmaceutical Co, Ltd, Tsukuba, Ibaraki 300-2611, Japan; 2 Corporate services, Banyu Pharmaceutical Co, Ltd, Chiyoda-Ku, Tokyo 102-8667, Japan

## Abstract

**Background:**

Wee1 is a tyrosine kinase regulating S-G2 cell cycle transition through the inactivating phosphorylation of CDC2. The inhibition of Wee1 kinase by a selective small molecule inhibitor significantly enhances the anti-tumor efficacy of DNA damaging agents, specifically in p53 negative tumors by abrogating S-G2 checkpoints, while normal cells with wild-type p53 are not severely damaged due to the intact function of the G1 checkpoint mediated by p53. Since the measurement of mRNA expression requires a very small amount of biopsy tissue and is highly quantitative, the development of a pharmacodynamic (PD) biomarker leveraging mRNA expression is eagerly anticipated in order to estimate target engagement of anti-cancer agents.

**Results:**

In order to find the Wee1 inhibition signature, mRNA expression profiling was first performed in both p53 positive and negative cancer cell lines treated with gemcitabine and a Wee1 inhibitor, MK-1775. We next carried out mRNA expression profiling of skin samples derived from xenograft models treated with the Wee1 inhibitor to identify a Wee1 inhibitor-regulatory gene set. Then, the genes that were commonly modulated in both cancer cell lines and rat skin samples were extracted as a Wee1 inhibition signature that could potentially be used as a PD biomarker independent of p53 status. The expression of the Wee1 inhibition signature was found to be regulated in a dose-dependent manner by the Wee1 inhibitor, and was significantly correlated with the inhibition level of a direct substrate, phosphorylated-CDC2. Individual genes in this Wee1 inhibition signature are known to regulate S-G2 cell cycle progression or checkpoints, which is consistent with the mode-of-action of the Wee1 inhibitor.

**Conclusion:**

We report here the identification of an mRNA gene signature that was specifically changed by gemcitabine and Wee1 inhibitor combination treatment by molecular profiling. Given the common regulation of expression in both xenograft tumors and animal skin samples, the data suggest that the Wee1 inhibition gene signature might be utilized as a quantitative PD biomarker in both tumors and surrogate tissues, such as skin and hair follicles, in human clinical trials.

## Background

A diversity of anti-tumor agents is known to cause DNA damage resulting in the activation of G1 and G2 cell cycle checkpoints [1-3]. Normal somatic cells with functional p53 arrest the cell cycle both at G1 and G2 phases by transactivating p53 regulatory genes upon DNA damage [4,5]. However, the G1 checkpoint is frequently compromised in multiple types of cancers due to loss-of-function mutations in the p53 gene [6,7]. Cancer cells with dysfunctional p53 are more reliant on the G2 checkpoint in order to repair damaged DNA. Wee1 kinase, which acts as a critical driver of G2-M cell cycle progression, is involved in S-G2 checkpoints through inactivating phosphorylation of CDC2 at the Y15 residue [8,9]. When DNA is damaged in cells, Wee1 is phosphorylated at S549 by several kinases, including CHEK1, followed by binding to 14-3-3 proteins which leads to stabilization of the Wee1 protein [10-12]. The phosphorylated and stabilized Wee1 increases the level of inactivated phoshorylated-CDC2, preventing the damaged cells from entering into premature mitosis without repairing the DNA. Although the activation mechanism is still controversial, various studies have established the essential function of Wee1 in the regulation of S-G2 cell cycle arrest in response to DNA damage.

Given the pivotal role of Wee1 in the S-G2 checkpoint, the inhibition of Wee1 kinase is expected to exert an anti-tumor effect by abrogating the G2 checkpoint, specifically in p53 negative tumors in combination with DNA damaging drugs. Several previous studies have illustrated the p53-context dependent anti-tumor efficacy of Wee1 inhibition *in vitro *[13-15]. A potent Wee1 inhibitor, PD0166283, sensitizes p53-negative cancer cells to radiation-induced cell death compared with p53-positive cells [13,14]. It was also shown that Wee1 silencing by siRNA potentiates the anti-tumor effect of Adriamycin in p53-defective HeLa cells, although normal mammary epithelial cells with wild-type p53 are not severely damaged [15]. Recently, we have developed a new class of small molecule Wee1 inhibitor as a G2 checkpoint abrogator, MK-1775 [16]. The Wee1 inhibitor induces cell death selectively in p53-negative cells compared with isogenic p53-positive cells in combination with DNA damaging agents such as gemcitabine, carboplatin, and cisplatin. The assessment of the primary substrate, phospho-CDC2, ensured that the p53 context-specificity was mediated by Wee1 inhibition. We also demonstrated that significant sensitization to various DNA damaging agents is observed in p53 negative xenograft tumors in rodents, providing the initial evidence that Wee1 inhibition enhances the effect of standard care medicine *in vivo *via abrogating the G2 checkpoint. Clinical development of the Wee1 inhibitor as a p53 context-specific sensitizer would potentially improve the low therapeutic indices and narrow therapeutic window from which current chemotherapeutic agents are suffering.

Development of pharmacodynamic (PD) biomarkers is critically important in cancer drug development in order to examine whether drugs are modulating the intended therapeutic targets or pathways [17-19]. Conventionally, immunohistochemistry (IHC) assays for protein-biomarkers have played an important role in assessing the target engagement level of drugs; such biomarkers include phosphorylated-EGFR for Iressa [20], and phosphorylated-CRKL for Gleevec [21]. For the Wee1 inhibitor, the phosphorylation level of CDC2 is a promising PD biomarker since it is a primary substrate for Wee1 kinase [14-16]. Indeed, reduction of phosphorylated-CDC2 at Tyr15 has been observed in both *in vitro *and *in vivo *studies, confirming that Wee1 inhibitors were engaging the target. Furthermore, the level of phosphorylation at Y15 is correlated with the anti-tumor efficacy of the Wee1 inhibitor. However, IHC assays for protein biomarkers have presented several challenges when developed in a clinical setting. First, IHC markers require a relatively large amount of biopsy tissue and morphological integrity, and these requirements are difficult to fulfill for some tumor biopsy methods, such as fine needle aspiration [22]. Second, IHC assays for proteins are not quantitative, since the expression level is usually indicated by the intensity scores of chromogens ranging from 0 to 3, which is a relatively arbitrary index. The development of mRNA gene expression signatures for anticancer drugs is an intriguing approach to overcome these drawbacks, since the measurement of mRNA requires smaller amounts of biopsy samples, and is highly quantitative when measured with an RT-qPCR assay. Multiple previous studies have measured mRNA expressions as PD gene biomarkers for estimating target engagement or predicting early response of anti-cancer agents such as KDR [23], COXII [24], or histone deacetylase inhibitors [25], providing evidence that mRNA gene signatures are suitable to quantitatively represent the indices.

The purpose of the present study was to develop a Wee1 inhibition gene signature measuring the change in expression caused by a combination treatment of Wee1 inhibitor and gemcitabine. Genome-wide gene expression in both cancer cells and skin tissues was analyzed to find a Wee1 gene signature that can be utilized in both tumor and surrogate tissues. The availability of the Wee1 gene signature in skin samples offers an advantage due to the difficulty of obtaining tumor biopsies from patients. In addition, dose-dependent expression changes of the Wee1 gene signature in rodent xenograft tumors and skin samples were correlated with the level of phosphorylated-CDC2 and anti-tumor efficacy of the Wee1 inhibitor. The expression pattern and function of the Wee1 gene signature are consistent with mode of action of the Wee1 inhibitor as a G2 checkpoint abrogator. These data ensure that the Wee1 gene signature identified in the present study can be utilized to assess the target engagement level of Wee1 inhibitor in both preclinical and clinical studies.

## Results

### Identification of Wee1 inhibition signature in cell lines

**Figure 1:**
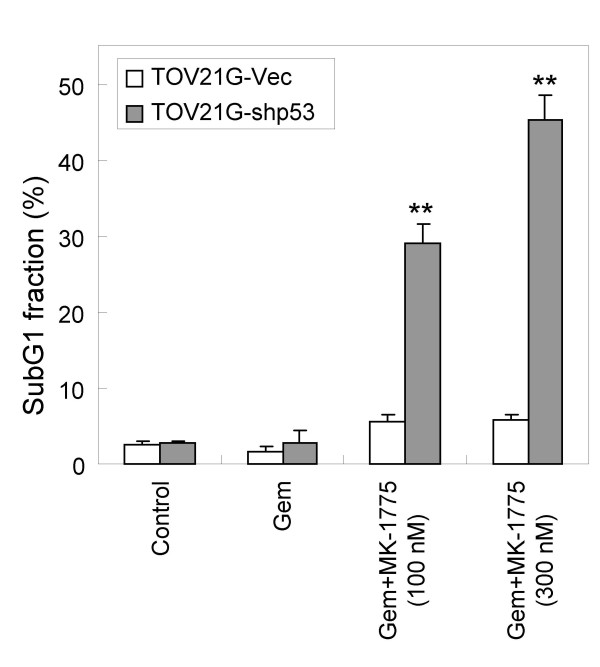
**TOV21G p53 positive and negative matched pair cell lines treated with gemcitabine and Wee1 inhibitor**. TOV21G positive- and negative cell lines were treated with 30 nM gemcitabine, and 100 and 300 nM of the Wee1 inhibitor. At 8 hr post Wee1 inhibitor treatment, cells were subjected to flow cytometry analysis. The percentage of the subG1 fraction corresponds to apoptotic cells. TOV21G-Vec: wild-type p53 cells; TOV21G-shp53: p53-deficient cells; Gem: gemcitabine. **, *P *< 0.01, compared with TOV21G-Vec.

**Figure 2:**
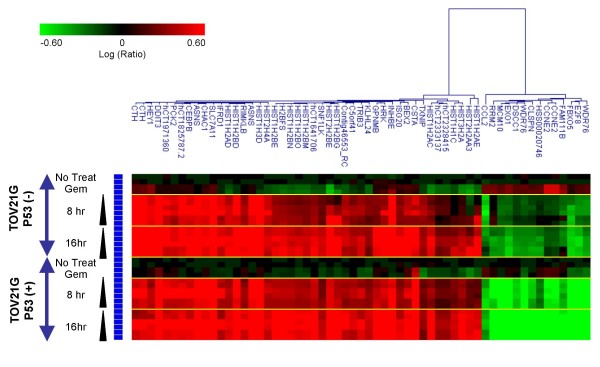
**Identification of a gene signature changed by gemcitabine and Wee1 inhibitor combination in both p53 positive and negative paired cell lines**. TOV21G positive and negative cell lines were treated with gemcitabine and Wee1 inhibitor as described in the legend of Figure 1, and mRNA from each treatment sample was applied to microarray analysis. A gene signature whose expression significantly changed in response to gemcitabine and Wee1 inhibitor treatment was extracted. Each row represents a sample from each treatment group. Each column represents a gene. Red, up-regulated genes; green, down-regulated genes.

We previously reported on a novel class of Wee1 inhibitor, MK-1775, (2-alkyl-6-anilino-1-aryl-1, 2-dihydro-3H-pyrazole [3, 4-d]pyrimidin-3-one) with an IC_50 _value of 5.2 nM against recombinant human Wee1 in *in vitro *kinase assays. MK-1775 potentiates the anti-cancer efficacy of DNA damaging agents such as gemcitabine, cisplatin, and carboplatin both *in vitro *and *in vivo *[16]. In order to find an mRNA gene signature that indicates target engagement of Wee1 inhibitor as a PD biomarker, we analyzed genome-wide expression profiles of p53-positive and -negative isogenic paired cell lines (TOV21G-Vec and TOV21G-shp53) treated with gemcitabine and Wee1 inhibitor. TOV21G is an ovarian cancer cell line with wild-type p53 gene. The isogenic pairs of p53-positive and -negative TOV21G cells were generated by transfection with a vector expressing an shRNA targeting p53 or an empty vector, respectively [26]. We employed p53 paired cell lines to find PD markers available in cancer cells independent of p53 status. First, gemcitabine was used to treat the p53 matched pair cell lines for 24 hr to activate S-G2 checkpoints. Next, increasing concentrations of MK-1775 were administered to the cells for 8 hr following the gemcitabine treatment. We confirmed that more significant apoptosis was induced in p53-negative cells compared with p53-positive counterparts in accordance with the previous study [16] (Figure 1). While 28% and 44% of the sub-G1 fraction was induced in p53 negative cells treated with 100 nM and 300 nM of the Wee1 inhibitor respectively, 5.9% and 6.4% of the sub-G1 fraction was observed in p53-positve cells. In parallel with the efficacy study, mRNA recovered at 8 and 16 hr after the Wee1 inhibitor treatment was subjected to microarray analysis to find the PD gene biomarker. We extracted genes whose expression levels in Wee1 inhibitor-treated cell lines were significantly up- or down-regulated compared to those of gemcitabine treated cell lines. We pared down the signature by extracting the genes whose expression exhibited greater than three-fold change in both p53 positive and negative cell lines in at least one treatment condition. A hierarchical clustering of the gene signature composed of 55 genes is shown in Figure 2, and the genes exhibited similar expressional regulation in both p53 positive and negative cells. Moreover, most of the genes showed time-dependent and concentration-dependent expression changes that are suitable features of PD biomarkers. Functional assessment of the gene signature by a hypergeometric test for gene enrichment indicated that S-G2/M cell cycle genes were significantly enriched in down-regulated genes (DNA replication: p = 8.4 × 10^-9^; S phase of mitotic cell cycle: p = 5.3 × 10^-8^; mitotic cell cycle: p = 1.69 × 10^-5^) and up-regulated genes (nucleosome assembly in M phase: p = 1.3 × 10^-23^). This finding is consistent with the function of Wee1 kinase that prevents premature mitosis entry.

### Identification of Wee1 inhibition signature in rat skin samples

**Figure 3:**
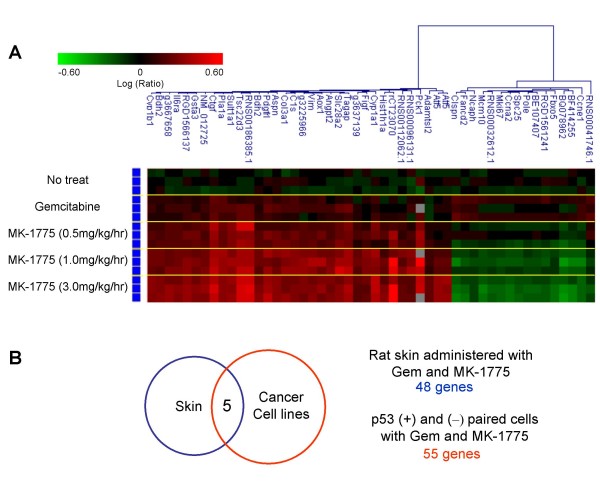
**Identification of a gene signature changed by gemcitabine and Wee1 inhibitor in rat skin sample**. (A) Expression profile of rat skin samples treated with gemcitabine and Wee1 inhibitor. Nude rats were administered with gemcitabine and increasing concentrations of the Wee1 inhibitor via IV infusion. At 8 hr post Wee1 inhibitor administration, mRNA from each rat skin sample was applied to microarray analysis. A gene signature whose expression significantly changed in response to gemcitabine and Wee1 inhibitor treatment was extracted. Each row represents a sample from each treatment group. Each column represents a gene. Red, up-regulated genes; green, down-regulated genes. (B) Wee1 gene signature commonly changed in both cancer cell lines and skin samples. The common signature for both tumor and surrogate tissues was identified by extracting commonly regulated genes in global expression profiling. Gem: gemcitabine; Wee1i: Wee1 inhibitor (MK-1775).

Although measuring PD biomarkers in tumors is preferable, skin is an attractive tissue since it is easily accessible for analyzing PD effects, especially for tumor types for which biopsies are difficult. In attempting to identify PD biomarkers in surrogate skin tissues *in vivo*, expression profiles were analyzed between rat skin samples treated with gemcitabine only and a gemcitabine/Wee1 inhibitor combination. Subcutaneous xenograft tumors were formed by injection of the human colorectal cancer, WiDr, in the hind flank of immunodeficient nude rats. On the 8th day, gemcitabine was intraveneously (IV) administrated to the animals. Twenty-four hours later, an increasing concentration of the Wee1 inhibitor was infused via IV infusion for 8 hr. Then, total RNAs from each rat skin tissue were purified and applied to microarray analysis to extract a gene signature whose expression significantly changed in response to gemcitabine and the Wee1 inhibitor treatment. The selection criteria to determine up- and down-regulated genes are described in the Materials and Methods in detail. Briefly, error-weighted ANOVA was applied between the Wee1 inhibitor-treated samples and gemcitabine treated samples, and the genes whose expression changed more than 1.5-fold in either 1.0 or 3.0 mg/kg/hr treatment were further selected down. As a result, 48 genes out of 39,558 probes were found to be significantly changed by gemcitabine/Wee1 inhibitor combination treatment compared with gemcitabine treatment only. Hierarchical clustering of the gene signature in rat skin is displayed in Figure 3 as a heatmap, showing the dose-dependent changes in their expressions.

### Extraction of Wee1 inhibition gene signature available in both tumor and skin tissues

To find genes that can be used as a PD biomarker in both tumor and skin tissues, a common gene signature that was changed in both cancer cell lines and skin tissue was extracted. In both experiments, claspin (CLSPN), minichromosome maintenance complex component 10 (MCM10), and F-box protein 5 (FBXO5) were significantly changed, indicating that they could be promising expression PD biomarkers for the Wee1 inhibitor independent of p53 status and the tissue type. CCNE1 was included in the gene set changed in skin samples, whereas CCNE2 was found in the analysis of p53 paired cell lines *in vitro*. Given the well-conserved function between CCNE1 and CCNE2, both genes were selected for the Wee1 inhibition gene signature for further validation. Previously reported functions of the five genes in the Wee1 inhibition gene signature which relate to the S-G2 cell cycle are shown in Table 1, inferring a relationship between Wee1 inhibitor-mediated gene expression changes and S-G2 cell cycle checkpoints.

**Table 1:** Function of Wee1 inhibition gene signature

**Gene**	**Function**
CLSPN	Clspn triggers a checkpoint arrest of cell cycle by activating CHEK1 in response to DNA damage
FBXO5	Fbxo5 is a mitotic regulator interacting with CDC20 and inhibits the anaphase promoting complex
MCM10	Involved in S phase progression which interacts with chromatin during S phase and dissociates G2
CCNE1	CyclinE1 forms a complex with and functions as a regulatory subunit of CDK2
CCNE2	CyclinE2 forms a complex with and functions as a regulatory subunit of CDK2

Although the 5 genes were selected as a common signature in both cancer and surrogate skin tissues, most of the cancer gene signature and rat skin signature showed statistically significant expression changes in reciprocal experiments, suggesting conserved Wee1-mediated expression changes in both tumor and the surrogate tissues.

### Validation of the Wee1 inhibition gene signature

**Figure 4:**
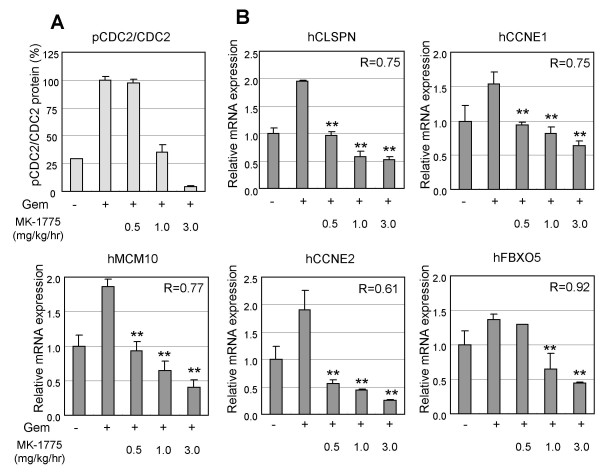
**Expression changes of Wee1 inhibition signature in xenograft WiDr tumors and their correlation to classical PD marker, pCDC2**. Human WiDr colorectal cancer cells were injected subcutaneously into nude rats. After allowing tumors to establish for 8 days, gencitabine and Wee1 inhibitor were administered in the nude rats as described in the legend of Figure 3. At 8 hr post Wee1 inhibitor administration, mRNA from each WiDr xenograft tumor was applied to quantitative RT-PCR analysis. To correlate the phosphorylated-CDC2 and Wee1 gene expression signature, the level of the pCDC2 normalized to total CDC2 is shown. The correlation coefficient (R) of pCDC2 expression and each mRNA expression is shown in each graph. Data represent mean ± SD. **, *P *< 0.01, compared with gemcitabine treated sample.

Expression changes of the Wee1 inhibition gene signature in cancer cells have thus far been assessed only in cultured cell lines. To validate the Wee1 inhibition gene signature, we analyzed mRNA expression of the five genes in WiDr xenograft tumors *in vivo*. With the same dosing regimen used in the rat skin microarray, nude rats bearing WiDr xenograft tumors were administered with gemcitabine and the Wee1 inhibitor combination. To analyze the gene markers, total RNA samples from the WiDr xenograft tumors were purified 8 hr after Wee1 inhibitor administration, and the expression of the Wee1 gene signature was measured by quantitative RT-PCR. As a result, the expression of all five genes was up-regulated by gemcitabine treatment, and subsequently down-regulated by the Wee1 inhibitor treatment, which was a similar expression pattern to that of TOV21G p53 matched pair cells *in vitro *(Figure 4). For example, gemcitabine treatment increased the expression of CLSPN by 2-fold, and Wee1 inhibitor down-regulated the expression to one-fourth compared with the gemcitabine single treatment sample. We also measured the level of phosphorylated CDC2 (Y15) in the WiDr xenograft tumor samples by Western blotting. The expression pattern of the Wee1 gene signature was similar to that of phosphorylated-CDC2 (Y15) when the correlation coefficient (R) was calculated between phosphorylated-CDC2 and mRNA expression of each gene in the Wee1 gene signature (Figure 4). This correlation supports the idea that functions of each gene in the Wee1 inhibition signature relate to the S-G2 cell cycle and/or its checkpoints. Regarding anti-tumor efficacy, statistically significant enhancement of efficacy for gemcitabine was observed, when co-treated with more than 0.5 mg/kg/hr (8 hr IV infusion) of MK-1775 (data not shown).

**Figure 5:**
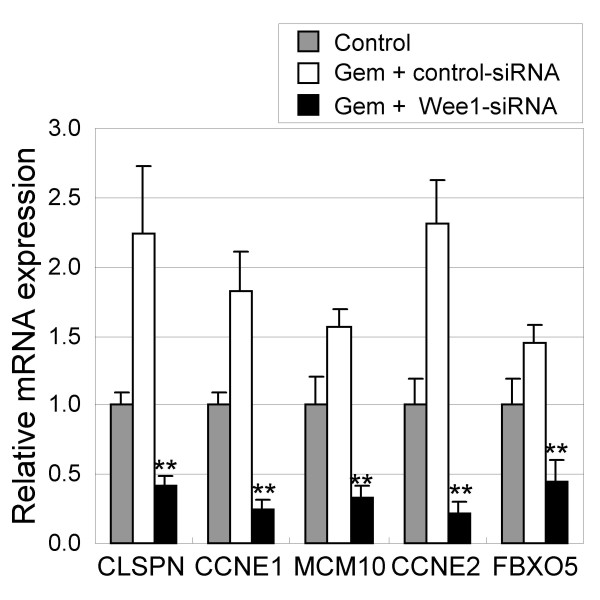
**siRNA-Wee1 treatment confirms that the selected genes are the *bona-fide *Wee1 inhibition gene signature**. WiDr colorectal cancer cells were treated with 30 nM of gemcitabine and siRNA for Wee1. After 48 hr siRNA treatment, mRNA from each was applied to quantitative RT-PCR analysis for the Wee1 gene signature. Gem: gemcitabine. Data represent mean ± SD. **, *P *< 0.01, compared with gemcitabine treated sample.

Finally, to confirm that the selected genes constitute a genuine Wee1 inhibition signature independent of the inhibition modality, the mRNA expression of the five genes were examined in WiDr cells treated with siRNA for Wee1 *in vitro*. Twenty-four hours after gemcitabine treatment, siRNA for Wee1 was transferred to the cells and the expression of the candidate signature was analyzed. In accordance with the results obtained in the Wee1 inhibitor study, significant down-regulation of mRNA expression was observed when Wee1 was silenced with siRNA (Figure 5).

## Discussion

A number of reports have shown the usefulness of protein biomarkers to assess target engagement of anti-cancer agents in tumors [20,21,27]. Some protein markers for the Wee1 inhibitor have also been reported in preclinical studies, including phosphorylated-CDC2 and -histone H3 [14-16]. Assays for protein-markers are in general not quantitative and require large amounts of biopsy specimens in clinical trials. The same holds true for protein markers for the Wee1 inhibitor. The development of a Wee1 gene signature as an mRNA-based expression biomarker offers some advantages over protein markers. The Wee1 gene signature presents quantitative data when measured by RT-PCR. This allows investigators to precisely correlate the changes in the expression of the Wee1 gene signature and anti-tumor efficacy of the Wee1 inhibitor. The Wee1 gene signature is also superior to conventional IHC markers such as phosphorylated-CDC2 in terms of the required amount of samples. To measure phosphorylated-CDC2 in cancer, several slices of formalin fixed paraffin embedded tissues (FFPET) are required for total CDC2, phosphorylated CDC2, and their confirmation assays. In contrast, one slice will be sufficient for multiple repeated measurements of the Wee1 gene expression signature. Since the quantification and amplification technologies of mRNA have been advancing rapidly [28,29], further reduction of required samples might be possible for analyzing the Wee1 gene signature.

In order to assess accurate target engagement of the Wee1 inhibitor, it is preferable to measure PD biomarkers in tumors. However, the feasibility of tumor biopsy is dependent on the tumor type [30,31]. While it is relatively easy to obtain tumor biopsies for skin cancers, biopsies of pancreatic or lung cancers are quite difficult. Therefore, the development of biomarkers that are commonly available in both tumors and surrogate tissues is of great benefit. Previous studies have proven that skin biopsies can be used to assess PD biomarkers of anticancer agents as an easily accessible tissue [32,33]. Although the development of mRNA gene expression biomarkers that can be measured in either tumors or surrogate tissues has been reported, the present study is unique in that the identified Wee1 gene signature can be commonly measured in both tumors and surrogate skin tissues. This was achieved by applying genome-wide gene expression profiling in the two tissues and extracting a commonly regulated gene signature. The Wee1 gene signature in surrogate skin tissues may accelerate the clinical development of the inhibitor by enabling biopsies for most patients at multiple time points.

The Wee1 gene signature is composed of five genes listed in Table 1. Although the method to identify the signature was a non-biased genome-wide approach, the function of each gene in the signature is closely associated with the mechanism underlying the Wee1 inhibitor-mediated S-G2 phase checkpoint abrogation. First, CLSPN is a cell cycle regulated protein whose expression peaks at S-G2 phases [34]. CLSPN interacts with CHEK1 kinase that also plays a pivotal role in the S-G2 cell cycle checkpoint, and association of the two proteins is required for CHEK1 activation in response to DNA damage [35]. Therefore, down-regulation of CLSPN expression by the Wee1 inhibitor would provide additional beneficial effects on S-G2 checkpoint abrogation by preventing the activation of CHEK1 kinase. Second, MCM10 is a DNA binding protein involved in the initiation of DNA replication as well as the elongation step [36]. Interestingly, it was reported that the depletion of MCM10 by small interfering RNA in cancer cells accumulates DNA damage and arrests the cells in late S-G2 phase, suggesting a role for MCM10 in cell cycle checkpoints [37]. We envision that DNA damage by gemcitabine arrested the cells in the S-G2 phase, which activates the DNA repair system in which MCM10 is involved. The abrogation of the S-G2 phase checkpoint by the Wee1 inhibitor might have reduced the expression of MCM10 without completion of DNA repair. Third, FBXO5, also known as Emi1, is a cellular inhibitor of the APC/C complex which degradates mitotic cyclins (Cyclin A and B) [38]. The up-regulation of FBXO5 ensures that the cells are arrested at S phase by gemcitabine, since FBXO5 inhibits APC/C during S phases. At the onset of mitosis, it is known that FBXO5 activity is significantly reduced [39], which could also explain the down-regulation of FBXO5 expression by Wee1 inhibitor. Finally, CyclinE1 and 2 are well-known regulators of S phase cell cycle progression [40]. Since the expressional regulation of CyclinE has extensively been investigated [41], the expression pattern found in this study was very reasonable. Similar to the hypothetical mechanism discussed for FBXO5, the expression pattern of CyclinE1/2 supports the mode-of-action of the Wee1 inhibitor that causes the disruption of S-G2 checkpoints leading to premature mitotic entry. Although we have speculated a functional relation between the Wee1 inhibitor and the gene signature, it would be interesting to further decipher the molecular role of the five genes in the Wee1 inhibitor-mediated anti-cancer effect.

**Figure 6:**
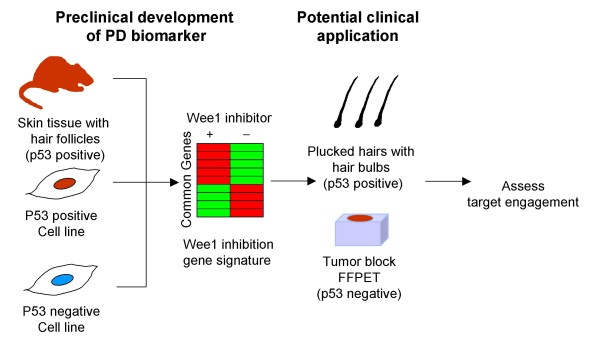
**Schematic diagram for finding the Wee1 gene signature available for both tumor and surrogate tissues, and its potential application to clinical research**. The present study isolated a gene set that commonly showed expression changes in both TOV21G p53 positive and negative matched pair cell lines and rat skin tissues, which led to the identification of the Wee1 gene signature available as a PD biomarker for Wee1 inhibitor independent of the p53 status of the tissues analyzed. In future clinical trials, the Wee1 gene expression signature can be analyzed from both plucked hair samples and FFPE tumor samples to assess the target engagement of the Wee1 inhibitor.

There are several challenges ahead before using the preclinically developed Wee1 inhibition gene signature in clinical trials. First, although the present data shows that the signature can be assessed as a PD biomarker in surrogate rat skin tissues, the signature should be evaluated in human surrogate tissues. Since the Wee1 gene signature is composed of cell cycle related genes, their expression changes should be observed in proliferating cells, which is also supported by the fact that actively proliferating tumor samples both *in vitro *and *in vivo *showed a larger effect size compared with rat skin tissues. As the actively growing cells in skin samples would be those from hair follicles or hair bulbs, a potential surrogate skin tissue utilized in human clinical trials is scalp punch biopsy, in which hair density is relatively higher compared with other parts of the skin [42]. Plucked hair, including hair follicles and hair bulbs, could be an alternative candidate RNA source for the Wee1 gene signature (Figure 6). It has been reported that plucked hairs can be leveraged as a source of PD markers for other cell cycle inhibitors [43]. Second, the variability of the Wee1 gene signature is unknown, which makes it difficult to judge whether the observed expression changes in the Wee1 gene signature are derived from the treatment effect, intrapatient variability, or natural decay of signal. One strategy to address these issues is to conduct phase 0 trials which are first-in-human studies performed before standard phase I trials are conducted [44]. The phase 0 studies may be designed to determine a statistically significant Wee1 inhibitor-mediated effect on the expression changes of the Wee1 gene signature. With the data from multiple time points both pre- and post-treatment with Wee1 inhibitor, the phase 0 study will provide us with variability data which will allow researchers to do a statistical power calculation for the PD effect for a future standard phase I study.

Despites several challenges for the future of the Wee1 gene signature, its assessment will have beneficial impacts on the development of the Wee1 inhibitor. The quantitative assessment of the signature will allow us to make early decisions even at dose-setting phase 1 trials by providing information on whether sufficient target engagement is achieved or not at tolerable doses.

## Conclusion

In this study, we identified a Wee1 gene signature whose expression was changed in response to a combination treatment of gemcitabine and Wee1 inhibitor. A common expressional regulation of the Wee1 gene signature was observed in xenograft tumor (p53 negative), cultured cancer cells (p53 positive and negative), and rat skin tissues (p53 positive). Although the signature was selected through genome-wide molecular expression, the functions of the genes are associated with S-G2 cell cycle checkpoint and their abrogation, which is also supported by the fact that the phosphorylated CDC2 level that represents the S-G2 checkpoint activation level is highly correlated with the expression pattern of the Wee1 signature genes. In addition to the common regulation of the signature genes independent of the tissue type and p53 status, Wee1-silencing by siRNA confirmed that the Wee1 gene signature is generally regulated by gemcitabine and Wee1 inhibition. The present study first found and validated the gene signature as a PD biomarker for Wee1 inhibitor, and also presented initial evidence that a common mRNA expression-based biomarker in tumors and surrogate tissues can be identified, which is an advantageous feature to facilitate anticancer drug development.

## Methods

### Cell culture

WiDr cell lines were obtained from the American Type Culture Collection, and were cultured according to the supplier's instructions. TOV21G p53-isogenic matched-pair cell lines were provided from ROSETTA INPHARMATICS [26], and were cultured with Dulbecco's Modified Eagle Medium (DMEM, Invitrogen).

### Flow cytometric analysis

Cells were first treated with 30 nM gemcitabine (GEMZER, Lilly) for 24 hr followed by addition of MK-1775 for 8 hr. Trypsinized single-cells were stained with propidium iodide with the CycleTEST plus DNA reagent kit (BD biosciences) and were analyzed in a FACS Calibur (Becton Dickinson) apparatus.

### Expression profiling of TOV21G p53 positive- and negative-matched pair cell lines

TOV-21G p53-isogenic matched-pair cell lines were treated with 30 nM gemcitabine for 24-hr, followed by addition of MK-1775. At 8-hr or 16-hr after MK-1775 treatment, cells were recovered for RNA extraction. Hybridization for microarray experiments was performed as follows: TOV21G-Vec, no treatment control (control) vs. TOV21G-Vec. No treatment (n = 2); Control vs. TOV-21G-Vec treated with 30 nM gemcitabine for 24 hr (n = 2); Control vs. TOV21G-Vec treated with 30 nM gemcitabine for 24 hr, followed by treatment with 100 nM, 300 nM, or 1000 nM of MK-1775 for 8 hr (n = 2 for each concentration of MK-1775); Control vs. TOV21G-Vec treated with 30 nM gemcitabine for 24 hr, followed by treatment with 100 nM, 300 nM or 1000 nM of MK-1775 for 16 hr (n = 2 for each concentration of MK-1775). The same hybridizations performed for TOV21G-Vec were also carried out for the TOV21G-shp53 cell line.

### Gene marker findings of *in vivo *WiDr xenograft nude rats

The PD gene biomarker was investigated *in vivo *in a WiDr nude rat xenograft model. Gemcitabine was dosed as an intravenous bolus (50 mg/kg). After 24 hr of gemcitabine administration, MK-1775 was dosed via intravenous infusion at doses of 0.5, 1.0, and 3.0 mg/kg/hr for 8 hr. Skin samples were isolated 8 hr after MK-1775 dosing. Hybridization for microarray experiments was performed as follows: Vehicle control pool (control) vs. Vehicle control self-reference (n = 3); Control vs. gemcitabine 50 mg/kg (n = 3); Control vs. gemcitabine 50 mg/kg with 0.5, 1.0, or 3.0 mg/kg/hr of MK-1775 for 8 hr (n = 3 for each concentration of MK-11775).

Total RNA from cultured cells or skin samples was isolated by using the RNeasy mini kit (Qiagen, #74104) with DNase I (Qiagen, #79254). Total RNA from skin or tumor tissues in rat xenograft model was isolated by Trizol reagent (Invitrogen, #15596-018), and the isolated RNA was repurified with an RNeasy mini kit. The purified RNA from each sample was converted to cDNA and hybridized to appropriate reference standards; rat skin microarray: three vehicle control samples; human cell line microarray: pooled TOV21G with control vector samples. Next, microarray analysis was performed with a Rosetta/Merck microarray, Human 44 k 1.1 and Rat 44 k 1.1. Expression profiles were analyzed by the microarray software, Resolver (Rosetta Inpharmatics) to identify the classifier genes for responder.

### Microarray data analysis

1) Rat skin sample: First, error-weighted ANOVA was applied between 1.0/3.0 mg/kg/hr MK-1775 treated samples and gemcitabine only treated samples, and the genes whose expression was significantly changed in both 1.0 and 3.0 mpk treatment (p ≤ 0.001) were extracted. Next, we selected genes whose expression changed more than 1.5-fold in either 1.0 or 3.0 mg/kg/hr treatment compared with gemcitabine only treated samples. Then, error-weighted ANOVA was applied between 3.0 mg/kg/hr MK-1775 treated samples and 0.5 mpk MK-1775 treated samples, and the genes whose expression significantly (p ≤ 0.05) changed were selected.

2) TOV21G-derived p53 matched pair cells: In each experiment of TOV21 p53 positive and negative cell lines, expression levels of MK-1775 treated cell lines were divided by those of untreated cell lines with the re-ratio algorithm in Resolver. (Calculated values were used for clustering). In each experiment of TOV21 p53 positive and negative cell lines, gene expression of MK-1775 treated cell lines were divided by those of only gemcitabine treated cell lines with the re-ratio algorithm in Resolver. (Calculated values were used for signature selection). After the re-ratio, signature genes, whose expression levels in MK-1775 treated cell lines were significantly up-or down-regulated compared to those of gemcitabine treated cell lines (p ≤ 0.01), were selected in all comparisons. Among the signatures, we further selected genes which exhibited greater than three-fold expression change in at least one condition in both vector and control samples.

For each set of the selected signatures, hierarchical clustering was done by the Rosetta Resolver system with cosine correlation and average link options.

### Quantitative RT-PCR Analysis

cDNA was synthesized from 1 μg of total RNA by using TaqMan reverse transcription reagents (PE Applied Biosystems, #N8080234). Quantitative real-time PCR assays for human CLSPN, CCNE1/2, MCM10, FBXO5, and GAPDH were performed in triplicate for cDNA samples in 96-well optical plates. Data were collected and analyzed using an ABI PRISM 7700 sequence detector system (PE Applied Biosystems). Primer and probe sequences for the quantitative RT-PCR are as follows: primers for CLSPN, 5'-AGGTGGAGGAAGGAGCGAA-3', 5'-TTTCCCCTGCTGTGCCAT-3'; Taqman probe for CLSPN, 5'-TGAACGAGAGCAGTGGCTTCGGG-3'; primers for CCNE1, 5'-AAATGGCCAAAATCGACAGG-3', 5'-TGCATTATTGTCCCAAGGCTG-3'; Taqman probe for CCNE1: 5'-CGGCGAGGGACCAGTGTGGG-3'; primers for CCNE2 by SYBR Green method, 5'-CTATTTGGCTATGCTGGAGGAAGT 3', 5'-TTCAGTGCTCTTCGGTGGTGT-3'; primers for MCM10 by SYBR Green method, 5'-CTCCAGATCCCAAAAGCTCATC-3', 5'-TGTTCCGAGAAATCGTCTGTAGG-3'; primers for FBXO5 by SYBR Green method, 5'-TGAAGAAGGTAGCCTCCTGGAG-3', 5'-TGGCAGCAAGTTTTTGTTGG-3'.

### Phosphorylated-CDC2 assay

Tumors were isolated 8 hr after MK-1775 dosing. CDC2 protein was solubilized by homogenizing cells in buffer containing 1% NP40, 0.1% Triton X-100, and was detected by Western blotting with an anti-p-CDC2Y15 specific antibody (Cell Signaling #9111). The captured antibodies were detected and stained with biotinylated anti-IgG and streptavidin/horse radish peroxidase. The immunostained area was quantified using Image Pro Plus software (Media Cybertics Inc.).

## Competing interests

The authors declare that they have no competing interests.

## Authors' contributions

SM conducted most of the experiments, performed data analysis, and drafted the manuscript. KY and HI performed microarray data analysis. TA performed the *in vivo *study, and TN performed the *in vitro *RT-PCR expression study. HH contributed to the design of the *in vivo *experiments and edited the manuscript. HK contributed to the entire design of the experiments. All authors read and approved the final manuscript.
